# Control of a Gel-Forming Chemical Reaction Network Using Light-Triggered Proton Pumps

**DOI:** 10.1021/acs.langmuir.4c04581

**Published:** 2025-03-19

**Authors:** Jacqueline Figueiredo da Silva, Ardeshir Roshanasan, Marcel Bus, Dimitrios Fotiadis, Armin W. Knoll, Jan H. van Esch, Heiko Wolf

**Affiliations:** † IBM Research Europe - Zurich, Säumerstrasse 4, 8803 Rüschlikon, Switzerland; ‡ Department of Chemical Engineering, Delft University of Technology, Van der Maasweg 9, 2629 HZ Delft, The Netherlands; ¶ Institute of Biochemistry and Molecular Medicine, University of Bern, 3012 Bern, Switzerland

## Abstract

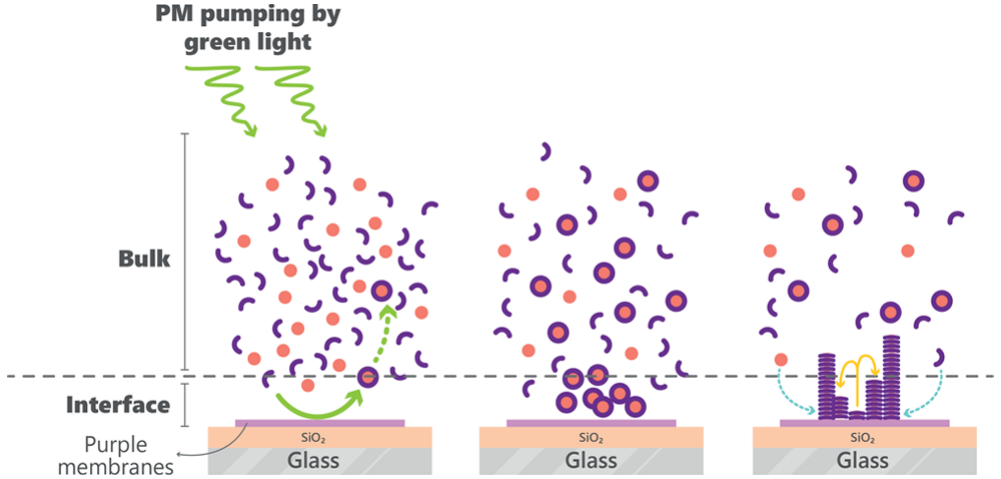

Numerous metabolic processes in nature are governed by extrinsic stimuli such as light and pH variations, which afford opportunities for synthetic and biological applications. In developing a multisensor apparatus, we have integrated submicrometer purple membrane patches, each harboring bacteriorhodopsin, onto a surface. Bacteriorhodopsin is a light-driven proton pump. We conducted monitoring of the interactions between this system and a pH-responsive supramolecular hydrogel to evaluate fibrous matrix growth. Initial photostimulation induced localized reductions in pH at the membrane surface, thereby catalyzing fibrogenesis within the hydrogel. Utilizing liquid atomic force microscopy alongside confocal laser scanning microscopy, we observed the hydrogel’s morphogenesis and structural adaptations in real time. The system adeptly modulated microscale pH environments, fostering targeted fibrous development within the hydrogel matrix. This elucidates the potential for engineering responsive materials that emulate natural bioprocesses.

## Introduction

Eukaryotic cells have evolved distinct compartments to accommodate the unique environmental requirements of diverse metabolic activities and facilitate energy storage using electrochemical gradients. Metabolism of fuels or harvesting of light are used to generate pH gradients,^1^ which are then exploited for a variety of key intracellular processes^2^ e.g. to maintain intracellular organization, transport molecules, and facilitate ion exchange between cellular compartments and with the environment.^3^ Hence, leveraging local pH variations as a stimulus for responsive systems has gained great interest for biomimetic applications and particularly in drug delivery systems. A similar prominence has been found in hydrogels^4^ which are also designed to respond to external stimuli.^5−9^ These hydrogels have been applied in controlled-release applications; for example, they act as ionic networks for the oral delivery of proteins,^10^ which is facilitated by the triggered collapse of the hydrogel. Alternatively, the hydrogel formation can be derived by a pH trigger. Although the formation of hydrogel by the means of a pH gradient has been demonstrated before,^11,12^ achieving rigorous, microscale spatiotemporal control over this process remains a challenge. Accordingly, our objective is to attain microscale regulation of the hydrogel formation via exposure to light as an external trigger, aiming to expand the applications of this material across various domains.

In nature, a microscale externally triggered pH gradient source is found in purple membranes (PMs). Bacteriorhodopsin (bR) is the main component of 5 nm thick PMs of *Halobacterium salinarum*.^13^ The bR acts as a light-driven proton pump, and the resulting pH-gradient is used for energy storage within the cells.^14^ Moreover, bR is a unique photochromic protein, and it has been successfully incorporated into various materials for the development of bio hybrid materials and nanostructured devices.^15,16^ The advantages of bR include broad absorption range of visible light, high thermal and photochemical stability, resistance to environmental perturbations, environmental friendliness, and the availability of genetic variants with enhanced spectral properties for specific device applications.^15^

Previous studies^3,13^ provide strong evidence that light irradiation directly drives a localized proton pump in PMs, mediated by the unique structural and functional properties of bR. When fabricating such hybrid devices, it should be noted that bR exhibits directionality in proton pumping and orientation within the membrane. The pumping occurs from the cytoplasmatic side (C-terminus) inside the cell to the extracellular side (N-terminus).^17^ Therefore, it is crucial to control the orientation of the membrane patches during deposition to optimize the photoelectric conversion efficiency. Through genetic modification, we generate a charge asymmetry between the cytoplasmic and extracellular sides of the PMs, which we use to control their orientation.^18,19^ Confocal laser scanning microscopy (CLSM), atomic force microscopy (AFM), and AFM-derived techniques such as single-molecule force spectroscopy (SMFS) have been combined to unveil information on PMs structure, functionality, and dynamics.^20^

Boekhoven et al.^5^ designed a Chemical Reaction Network (CRN) driven by a synthetic self-assembled system featuring a supramolecular hydrogelator. The CRN shows directed molecular self-assembly dynamics, where catalysis modulates reaction rates, impacting fiber morphology and promoting branching to form a dense gel network. The two-stage assembly—initial fiber formation followed by branching— yields gels with tailored mechanical properties. By encompassing kinetics-driven assembly, nonequilibrium states, and reversible hydrazone chemistry, the CRN demonstrates how catalysis governs material properties, transitioning from building blocks to functional hydrogels through a dynamic and tunable pathway. This hydrogelator not only regulates the self-assembly rate but also the properties of the resulting materials via bulk catalysis in aqueous medium, with a formation rate that can be tuned in situ by acidic catalysis. Examples of surface-confined catalysis that provide spatial control over the gelation process without external stimuli have been demonstrated, including microscaled surface-confined catalysis,^21^ protonated polymer brushes,^22^ and charged catalytic nanoparticles.^23^ To introduce external control, a photoswitchable homogeneous catalyst for light-activated gelation process was investigated.^24^ Building on these advancements, this study presents a novel approach that leverages light-driven metabolic proton pumping to locally trigger gelation.

In this work, a bicomponent system has been fabricated, comprising localized light-responsive proton pumps coupled with a pH-responsive hydrogel-forming CRN. The transient local proton gradient, sustained by illumination, facilitates confined material formation. Specifically, we exploit PMs to produce local light-driven proton gradients via incorporating them into miniaturized systems, thus enabling the localized control of proton-catalyzed hydrogel formation. We detected a pH decrease in a microscale area indicated by an irreversible fiber growth locally accelerated by protons. The direct influence of PM pumping on the microscale hydrogel formation over time was demonstrated in situ by liquid AFM and CLSM. The approach can be used to characterize the formation of the hydrogel at the microscale under relevant conditions.

## Experimental Section

### General Remarks

All reagents were purchased from commercial sources and were used as provided unless otherwise stated. Triacyl hydrazide cyclohexane and benzaldehyde were prepared according to published procedures.^25^ Liquid AFM data visualization, and data analysis were conducted using Gwyddion.^26^ CLSM data were processed using ZEN 2011 (Carl Zeiss) and ImageJ.^27^ All imaging was performed at room temperature.

### Preparation of Glass Substrates for Spatiotemporal Control of Gel Formation

Before use, glass substrates (24 mm × 24 mm) were treated with acetone and isopropanol, and the samples were treated with a plasma cleaner. The thickness of the glass chips was 170 μm for CLSM and liquid AFM. SiO_2_ was deposited (100 nm) on top of the glass chips via plasma-enhanced chemical vapor deposition (PECVD). In the dark, 12.5 μL of dispersed PM, either wild-type (WT), or the genetically modified C-His_10_-tag (bR with a deca-his tag at the C-terminus), or N-His_10_-tag (bR with a deca-his tag at the N-terminus), was added to the substrates in water at a concentration of 10^–2^ mg·mL^–1^. The deca-histidine His_10_-tag attachment to either the cytoplasmatic side (C-His_10_-tag) or extracellular side (N-His_10_-tag) of the PM was used to increase a charge asymmetry.^18,19^ The engineering, production and isolation of the PM versions are described in detail elsewhere.^18^ The samples were dried in the dark under ambient air, rinsed with Milli-Q water, and then dried under a nitrogen stream.

### Confocal Laser Scanning Microscopy

In the CLSM experiments, a PM-coated glass was carefully placed on a polydimethylsiloxane (PDMS) cuvette (12 mm × 12 mm × 3 mm) containing gelator precursors in a phosphate-buffered saline (PBS) at pH 7.0.^21^ The PMs were facing the solution. The two elements were then flipped and placed on the microscope. A fluorescein-based aldehyde-labeled probe (prepared according to the process described in ref (5).) was used at 0.01 molar percentage. Samples were monitored using a Zeiss LSM 980 equipped with a Zeiss Axio Observer inverted microscope, and Plan-Apochromat 63x oil immersion objective lens (NA 1.4). Incident wavelengths of 488 and 543 nm were used to visualize the gel formation and PM pumping, respectively; and emission at 517 nm was recorded. A z-step size of 48 μm was used to optically section the samples to seven planes. Snapshots of z-stacks were acquired every 67 s using a 488 nm laser, after which the sample was exposed to a 543 nm for PM pumping with the same time exposure. The 8-bit images of the middle plane were used for analysis.

### Liquid Atomic Force Microscopy

We operated a commercial Dimension-Icon AFM (Bruker)(Figure S2a, Supporting Information (SI)) equipped with triangular oxide-sharpened silicon nitride (Si_3_N_4_) cantilevers (length: 70 mm; ScanAsyst Fluid^+^) in ScanAsyst mode. Images were obtained from cantilevers under 2 kHz oscillation frequency, 3 Hz scanning rate, and 150 nm amplitude. An area of 3 × 3 μm^2^ was scanned at 128 × 128 samples/line. After the photodiode signal was stabilized, we began scanning the area again at a rate of 1 scan/min for 1 h. For all samples, we immersed the glass substrate in 140 μL of PBS buffer (100 mM, pH 7.0), and then imaged an area containing at least one PM patch. Subsequently, we injected 140 μL of gelator precursor solution onto the substrate at pH 7.0, at a concentration presented elsewhere.^5^ The experiment time is initiated from this point. One hour after the addition of gel chemicals, an area of 5 × 5 μm^2^ was scanned at 256 × 256 samples/line to check for any tip-induced desorption of gel agglomerates. For illuminating the scanning substrate with green light, we utilized a customized stage comprising a chip holder and a light-emitting diode (LED) green light source (530 nm) with varied intensities positioned beneath the chip holder, as depicted in Figure S2b, Supporting Information. The intervals and duration of illumination were manually controlled using the T-CubeLED Driver (ThorLabs). All experiments were performed with the same light power. AFM heights were measured by analyzing at least two PM patches. The average height difference was calculated within a 10-pixel region surrounding the height difference line, and the final average height was determined from these measurements.

## Results and Discussion

**Figure 1:**
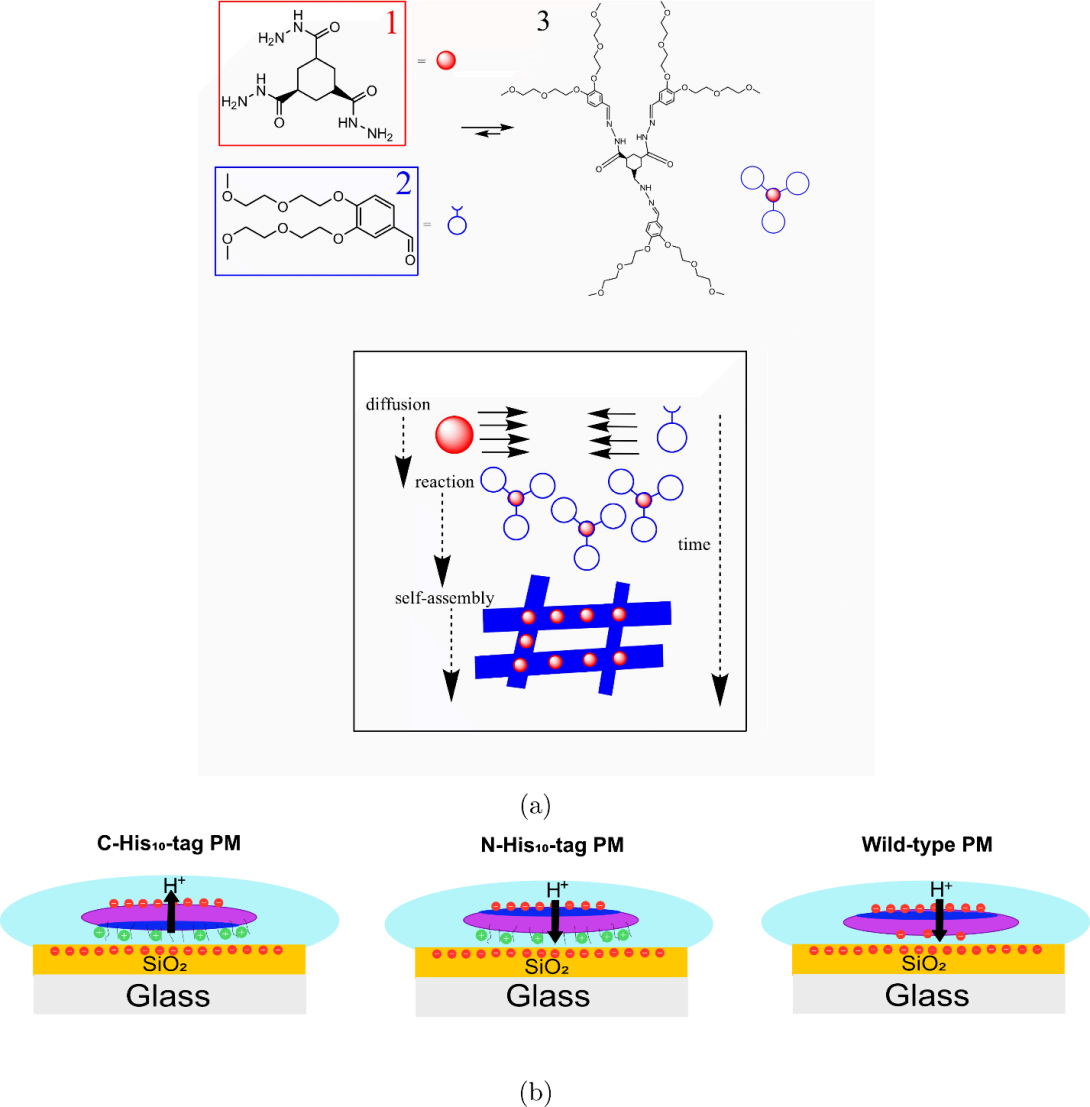
(a) Catalytic formation of tris hydrazone hydrogelator **3** from soluble building blocks **1** and **2**, leading to fiber formation by self-assembly and, subsequently, to a network of fibers that trapped the surrounding solvent to form a gel network. (b) Charge distribution on the SiO_2_ surface and PMs in aqueous media for C-His_10_-tag, N-His_10_-tag, and wild-type PMs and the respective direction of proton pumping. The purple, navy blue, light blue, red, and green areas represent PMs, the cytoplasmatic side of PMs, aqueous media, negative charge, and positive charge, respectively.

The system developed in this work includes a substrate containing PM patches for generating a local pH gradient under light, and a pH-triggered hydrogel system for detecting this gradient. The hydrogel system consists of the acid-catalyzed reaction of cyclohexane-1,3,5-tris-carbohydrazide **1** with three molecules of 3,4-bis[2-(2-methoxyethoxy)ethoxy] benzaldehyde **2** to form the actual hydrazone hydrogelator **3**, which subsequently self-assembles in water to form fibers.^5,25,28^ Above a certain concentration threshold, these fibers form a network in the aqueous phase, thereby leading to the formation of hydrogels,Figure 1a.

The PM part of the system is constructed from PMs that are deposited on SiO_2_ substrates. Previously, it was documented that PMs can adhere to SiO_2_.^19^ To control the orientation of PM adsorbed to SiO_2_ substrates, we have used three types of PMs: WT PMs, and two genetically modified PMs, namely N-His_10_-tag and C-His_10_-tag PMs. As shown in Figure 1b, in aqueous media, SiO_2_ layers present a negatively charged surface, whereas His_10_-tag peptide sequences impart a positive charge on the designated side of the PM (C-terminus or N-terminus).^18^ Thus, given the asymmetric positive surface charge on the extracellular side of the membrane juxtaposed with the negative charge of SiO_2_ layer in water,^29^ the N-His_10_-tag PMs are expected to preferentially pump protons from the PM-aqueous interface to the substrate–PM interface. Therefore, for N-His_10_-tag PMs we anticipate a photoinduced proton gradient toward the SiO_2_ substrate. The C-His_10_-tag PMs, on the other hand, should preferentially pump protons from the substrate–PM interface across the PM membrane toward the PM-aqueous interface, thereby creating a proton gradient oriented predominantly away from the SiO_2_ substrate. WT PMs have fragments with net negative charge on both sides of the membrane because of the amino-acid residues on the bR surface and the intrinsic acidity of PM lipids. However, at pH > 5 the PM surface charge density is more negative on the cytoplasmic side than on the extracellular side.^17^ Consequently, the orientation of the WT PM fragments is expected to be akin to that of N-His_10_-tag PMs.

The preparation of substrates for the experiments was carried out as described in the experimental section. Successful deposition of PMs across all types was confirmed by AFM imaging under ambient conditions, as shown in Figure S1, Supporting Information. Further detailed analysis revealed variations in the density of PMs on the SiO_2_ substrate surface, with N-His_10_-tag PMs exhibiting the highest density, followed by WT PMs, and finally C-His_10_-tag PMs. These differences are attributable to the interplay between the surface charge properties of SiO_2_ and those inherent to the PMs.^19^

We have used these PM-SiO_2_ systems in combination with the hydrogel-forming CRN to investigate surface-confined hydrogel formation. Microlocalized light exposure and higher imaging resolution were utilized in order to understand the effect of proton pumping orientation on the catalytic process. Our study incorporates experiments using CLSM for microlocalized excitation and compares these findings with liquid AFM offering submicrometer resolution. This complementary methodology facilitates a thorough analysis of the mechanisms responsible for the in situ formation of nanoscale hydrogels triggered by external stimuli.

### Spatiotemporal Control of Hydrogel Formation at the Microscale via Confocal Laser Scanning Microscopy

In a demonstration for in situ, externally triggered, microlocalized hydrogel formation, we explored whether a PM-coated surface could catalyze the formation of hydrogels confined to a surface. The preparation of the substrate involved covering a PM-coated glass surface with a solution containing hydrogel precursors in a buffered environment at pH 7.0. This minimizes the likelihood of premature gelation in the bulk.^25^ A detailed observation of a selected area on the PM-functionalized glass substrate was conducted to examine the aggregate formation resulting from light exposure. In contact with solution, the area was exposed to cycles of monochromatic excitations, alternating between wavelengths of 488 and 543 nm, each for approximately 1 min. The former was used for image acquisition at 517 nm, and the latter was implemented to induce proton pumping. Aiming at scanning the sample prior to expected gelation, the aforementioned light exposure cycle commenced with image acquisition. A blank glass substrate that was not coated with PM served as a control and was subjected to the same experimental procedure, which granted access to a comparative analysis to distinguish the effects and changes attributable to the presence of PMs.

**Figure 2:**
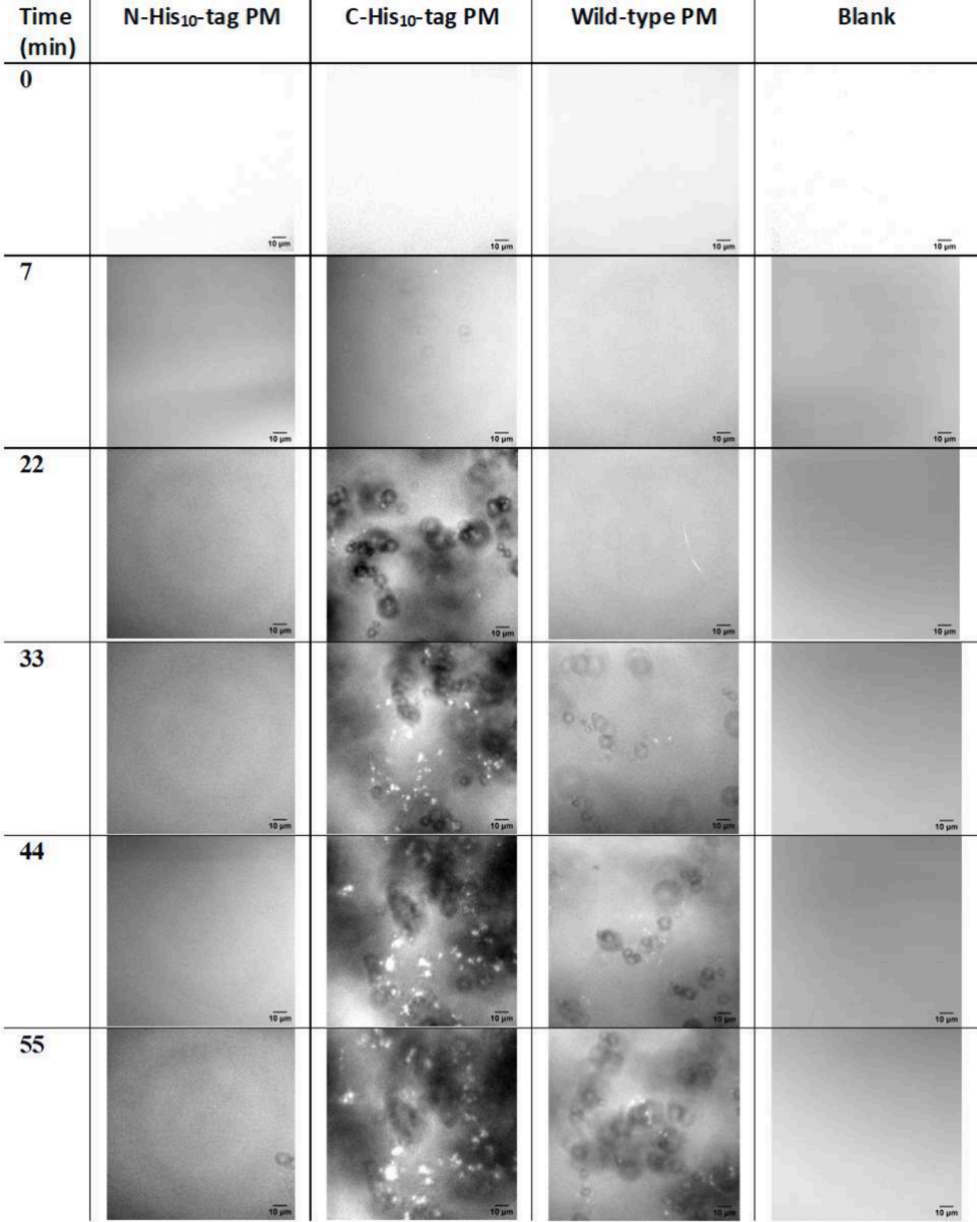
Confocal laser scanning microscopic images of the progression of hydrogel formation for different types of PMs and in the absence of PMs (blank). Snapshots were taken at 0, 7, 22, 33, 44, and 55 min at 63× magnification.

CLSM images in Figure 2 present the time progression of gel reactants, where different types of the PMs are present, in comparison to a blank sample in absence of PMs. We noted the onset of a novel phase characterized by globular cluster formation, that further coalesce to form larger domains. This phase does not form in the blank sample. Moreover, the gelation occurs in different time scales depending on the type of PMs; while the gel clusters start appearing for C-His_10_-tag PM under 7 min and reach a steady state within 44 min they only start forming after 22 and 55 min for WT PMs and N-His_10_-tag, respectively. This earlier aggregation likely resulted from the formation of a favorably aligned local pH gradient at the substrate surface due to PM orientation. Previous work^5,25^ using turbidity measurements demonstrated that gelation developed much faster in catalyzed samples than in uncatalyzed ones. The acid-catalyzed sample reached maximum absorbance within 60 min, while the uncatalyzed sample took approximately 9 h. These findings confirm that gelation is significantly slower in a pH 7 buffered medium without a catalyst, consistent with our results.

Interestingly, repeated experiments indicated that gel formation for the C-His_10_-tag PM-coated substrate can occur even prior to exposure to green light (543 nm) intended for pumping. It should be noted, however, that the samples already have been exposed to blue light (488 nm) to acquire the images at t = 0 min. This suggests excitation of the PM by 488 nm already leads to proton pumping. Previous work^30^ on PMs reported that electrical signals due to PM pumping peak at approximately 560 nm, but still have a significant response at 488 nm, which corroborates this lack of a selective pumping response. It is worth mentioning that the gelation was not observed in samples with N-His_10_-tag and WT PMs in the initial images, but only at significant later stages.

In summary, the presence and type of PMs significantly influenced the gelation rates. Specifically, C-His_10_-tag PMs, that are expected to pump protons from the SiO_2_ substrate–PM interface to the PM–aqueous interface, promote aggregates directly adjacent to the PM-aqueous interface, presumably by creating a favorably aligned pH gradient. In contrast, for WT-PM patches and N-His_10_-tag PMs, the gel formation is significantly slower. While the supposedly unfavorably aligned pH gradient generated by N-His_10_-tag PMs exhibits the longest formation time, the unsystematical alignment in WT-PMs yields a much shorter formation time. The most likely explanation is that their relatively reversed orientation does not lead to the same pH decrease near the PM-aqueous interface upon pumping. The C-His_10_-tag PM-induced pH gradients from the surface toward the bulk solution accelerate formation of the bicomponent hydrazone gelator,^25^ thereby increasing its local concentration and facilitating the gelation close to the surface. Therefore, the gelation rate is affected by the pH gradient generated by different PM types.

The developed system encouraged further analysis. Although the observations were aligned with our expectations, the limited wavelength selectivity of PM excitation and proton pumping during CLSM measurements asked for additional evidence. Therefore, another set of light-induced gel formation experiments were conducted, where in situ liquid AFM for image acquisition in the dark in combination with excitation of the PM by an independently operated light source were utilized.

### Atomic Force Microscopy for Capturing Temporal Control over Gel Formation at the Nanoscale

Liquid AFM was used to investigate nanoscale hydrogel formation influenced by PM and light near PM-covered SiO_2_ surfaces. The conditions for light exposure diverged from those utilized in CLSM as the setup enables image acquisition without irradiation, thereby counteracting the limited wavelength selectivity of CLSM in imaging and pumping. Thus, it was possible to separate pumping and imaging steps, while observing structure formation directly at the PM-aqueous interface for individual PM patches by employing multiple periods of green light exposure (543 nm) interspersed with dark periods.

**Figure 3:**
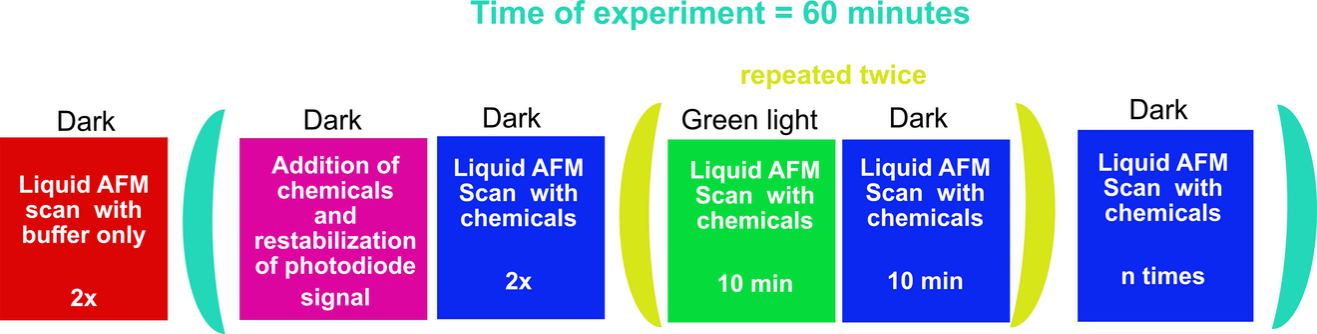
Diagram of the protocol of AFM experiments, including irradiation sequences and AFM image acquisition: red, AFM scan without gel reagents; pink, procedure without a scan; blue, AFM scan of a sample after the addition of chemicals in the dark; green, AFM scan of a sample after the addition of chemicals under green light.

Figure 3 shows the protocol of the AFM and irradiation experiments. Initially, the AFM scan was conducted in the absence of light on a surface coated with PM, which was covered with a buffer at pH 7.0 devoid of any gelator precursor molecules. After two scans, a solution of the gelator precursor molecules was added to the buffer. The final concentrations of gelator precursor reagents were half of those used in CLSM experiments. This reduction was crucial to prevent contamination of the AFM cantilever prior to scanning. Because of the change in liquid refractive index and temperature fluctuations due to addition of reagents, the photodiode signal was unstable for a few minutes (from 4 to 10 min). After the signal stabilized, the image acquisition-irradiation sequence was continued. For all PM types, the scan position remained nearly unchanged, except for C-His_10_-tag PMs, which shifted to a different area. We scanned two images in the dark, after which we irradiated with green light using a light-emitting diode (LED) localized at the back of the substrate (Figure S2b) for 10 min while scanning. Next, the sample was scanned in the dark for 10 min, and again for another 10 min upon exposure to green light. Finally, the substrate was scanned repeatedly until 1 h after addition of the gelator precursor reagents. Subsequently, a larger scan of 5 × 5 μm^2^ was performed to check for any influence of the tip scan on the structure formation at the surface. For C-His_10_-tag PMs, this investigation was not possible because the gel had already formed around the entire substrate area before the end of the experiment, contaminating the AFM cantilever with fiber deposits and preventing any further scans.

First, a PM-free substrate (Figure S3, Supporting Information) was subjected to scanning as described in Figure 3 as a control experiment. As expected, the AFM scan of the PM-free substrate remained a flat homogeneous surface without any new objects appearing up to 1 h after the addition of gel reagents, even for a large scan area (Figure S4, Supporting Information). Therefore, we concluded that in the PM-free samples, fibers were not formed on the substrate surface in the dark, nor upon irradiation with green light.

**Figure 4:**
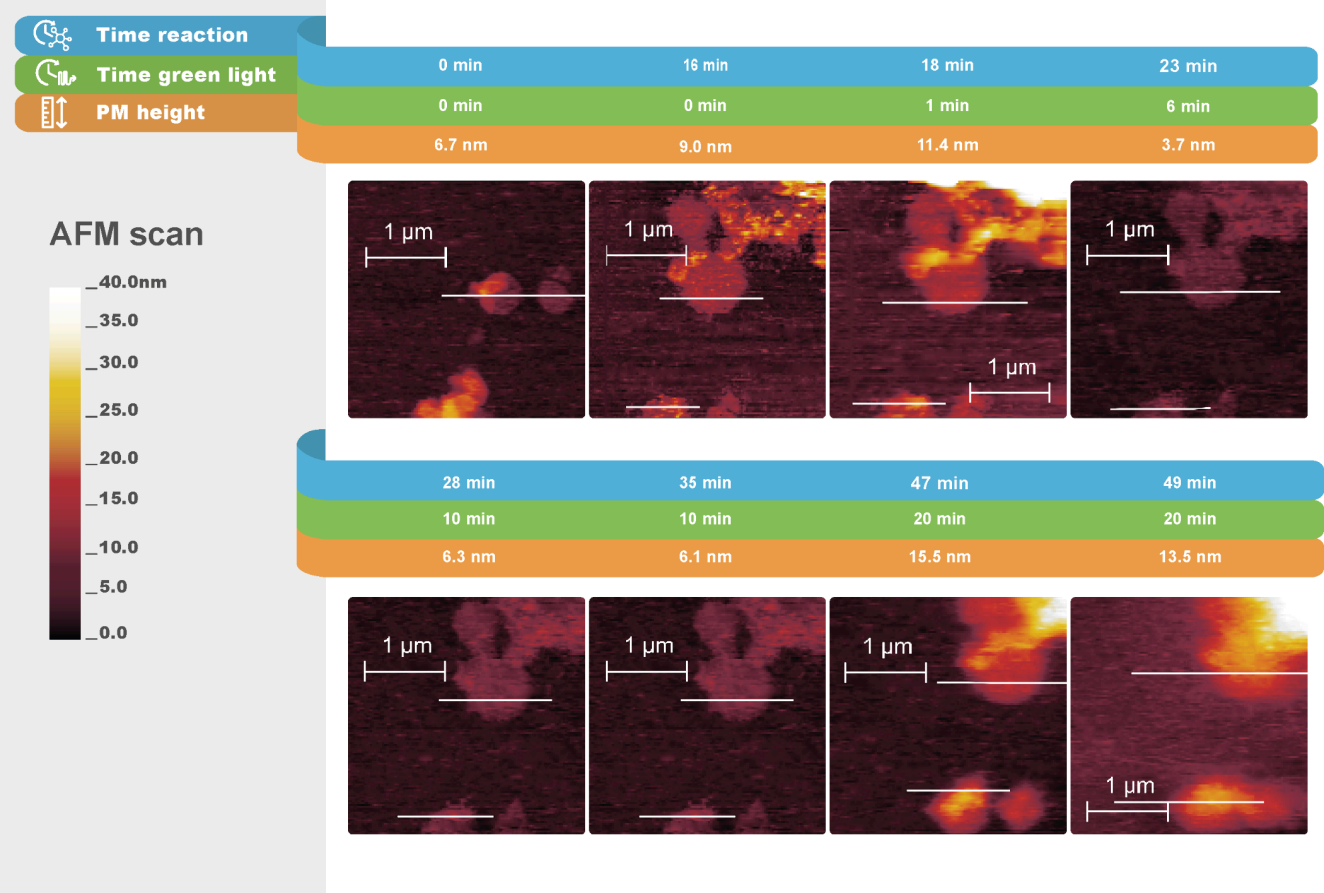
In situ AFM topography images (peakforce tapping mode in fluid) of hydrogel formation under the influence of PMs (C-His_10_-tag PMs) and light at different times. The lines highlighted in white indicate the cross sections of PMs. The scan direction is from right to left, with a 0° scan angle. Cross section graphs are shown in Figure S5, Supporting Information.

**Figure 5:**
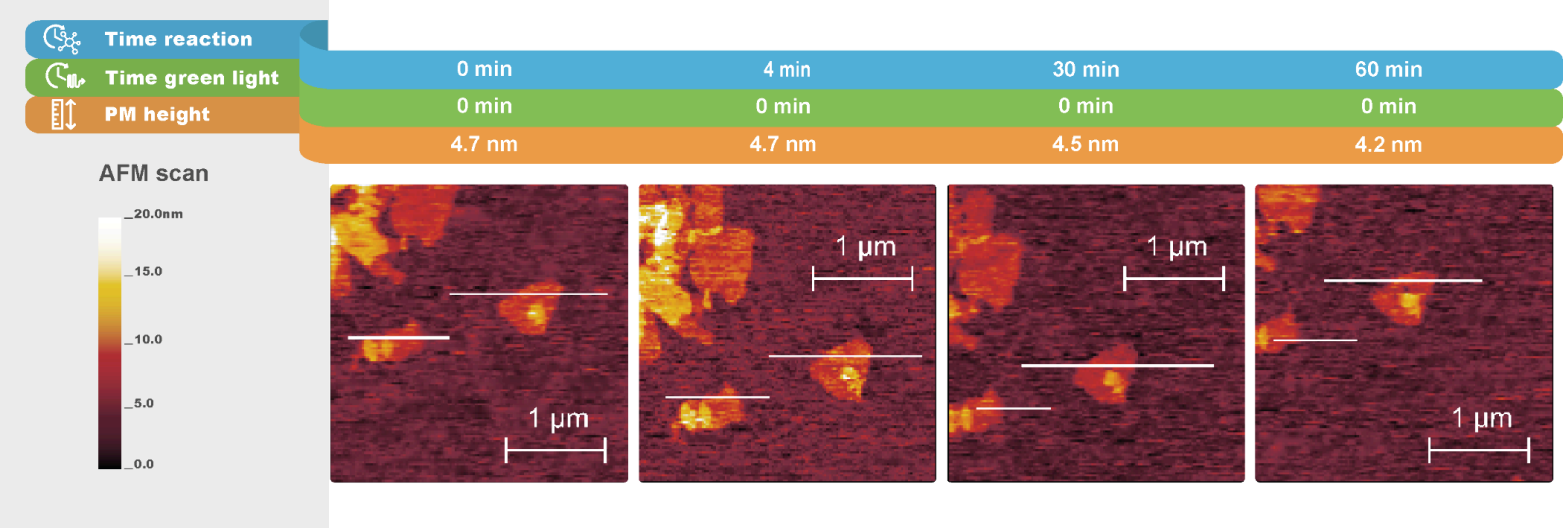
In situ AFM topography images (peakforce tapping mode in fluid) of a C-His_10_-tag PM-covered substrate kept in the dark for different times. The lines highlighted in white indicate the cross sections of PMs. The scan direction is from right to left, with a 0° scan angle. Cross section graphs are shown in Figure S6, Supporting Information.

In a subsequent series of experiments, we studied the substrates covered with the different types of PM. For all PM types, the AFM scans before the addition of reagents showed ellipsoidal ∼500 nm wide homogeneous patches with a height of approximately 5 to 8 nm on an otherwise flat surface (Figure 4, Figure 5, Figure 7 and Figure 8, Time = 0 min) that we assigned to PMs adsorbed at the interface. The maximum force at which the AFM tip scanned the membrane was limited to ∼100 pN to prevent mechanical deformation of bR,^31^ and the parameters of the AFM feedback loop were optimized to reduce error signal.^32^

Given the CLSM results, we anticipated a rapid gel formation soon after the green light exposure in the AFM scan of C-His_10_-tag PM, shown in Figure 4. At the onset of the experiment, during injection of the chemicals and stabilization, the lateral sample position shifted and thus two other PM patches were chosen for monitoring the fiber formation under exposure to green light (Figure 4, Time = 0 min and Time = 16 min). The PMs have an average height of 9 nm relative to the substrate. After a 1 min exposure to green light, the relative height of the PM area increased from 9.0 to 11.4 nm. Subsequently, while still under light, the relative height of the PM area decreased to 3.7 nm. During the time in the dark (between Time = 28 min and Time = 35 min), no change in the patch height was observed. From 10 to 20 min of exposure to green light, the height increased again to 15.5 nm. It is hypothesized that fiber networks initially form locally on the patches. The initial increase in the height of the patches of approximately 2.5 nm under illumination is consistent with the height of fibers reported in previous studies.^21^ Subsequently, a reduction in the relative height of the patches is noted. Localized gel formation is observed when the sample is subjected to green light once more, with the patch height increasing from 6.1 nm (Figure 4, Time = 35 min) to 15.5 nm (Figure 4, Time = 47 min). Ultimately, the hydrogel domains start to coalesce, and fiber formation is observed throughout the scanned area. The results suggest that initially the fibers grew preferentially on the PMs when exposed to light; subsequently, owing to proton diffusion, growth occurred in other areas as well. This indicates the light-triggering influence within the system. To demonstrate this assumption, we compared these results with a C-His_10_-tag PM-coated substrate scanned by AFM for 1 h in the presence of gel reactants in the dark (Figure 5). For this sample, the formation of fibrillar structures of gel patches was not observed. Therefore, we concluded that gel fiber formation is driven by the presence of both, C-His_10_-tag PMs and light.

**Figure 6:**
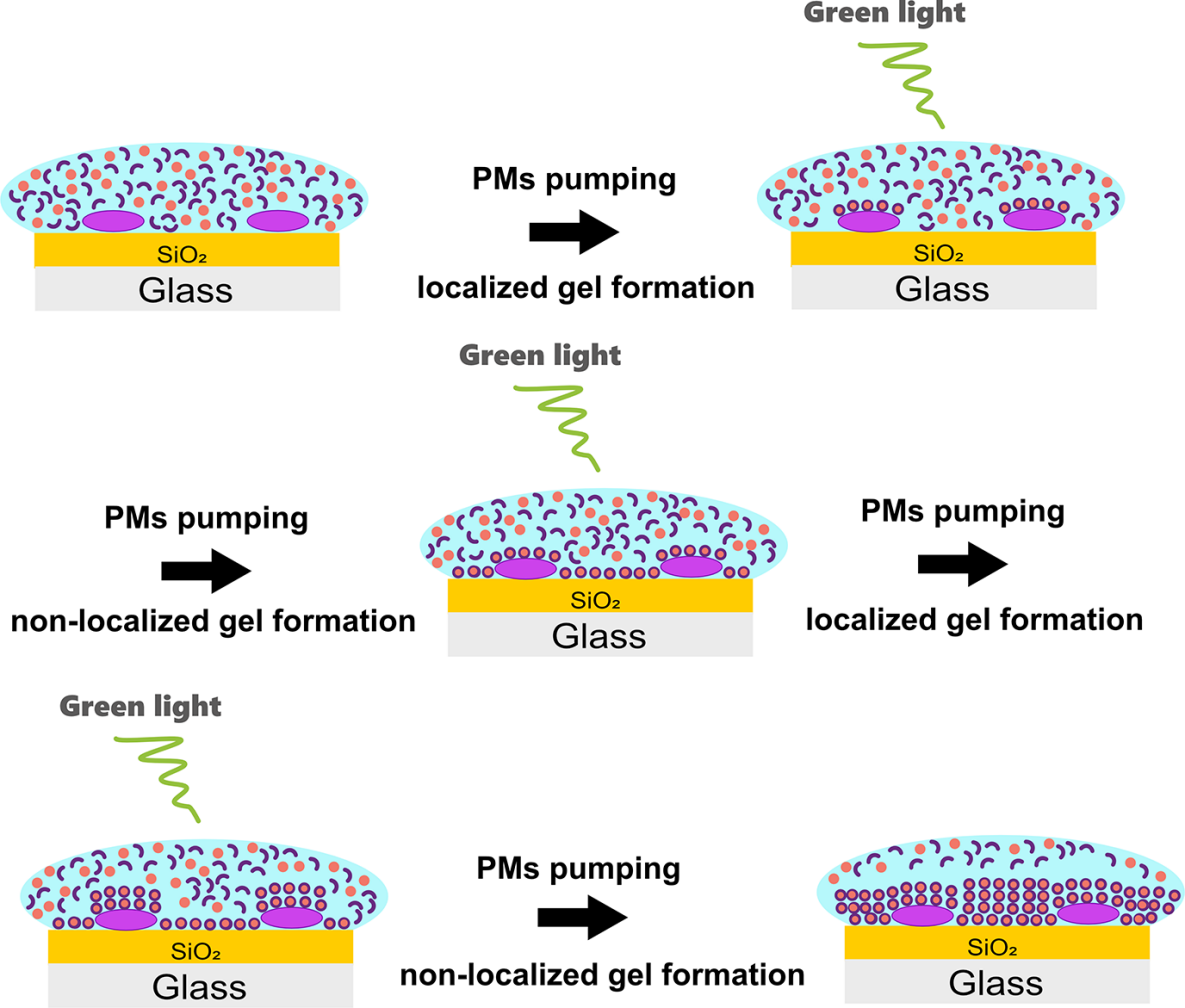
Time-lapse illustration of hydrogel formation after contact with C-His_10_-tag PM and green light observed by liquid AFM. The purple, light blue, dark blue, and red areas represent PMs, aqueous media, hydrazine molecules, and benzaldehyde molecules, respectively. The fiber is formed when three hydrazine molecules react with benzaldehyde.

Figure 6 illustrates the mechanism of gel formation under the influence of PMs and light, specifically observed for C-His_10_-tag PM. This process initiates with the acid-catalyzed transformation of precursor molecules into the active gelator. Then, when the local concentration of gelator molecules exceeds the critical gelation concentration, gel fibers start to form on the PM patches. During this process, protons diffuse into the bulk, resulting in acidification and the formation of fiber networks across the substrate. However, a localized gel formation still proceeds. Finally, a fibrous network is observed throughout the substrate owing to the proton diffusion.

**Figure 7:**
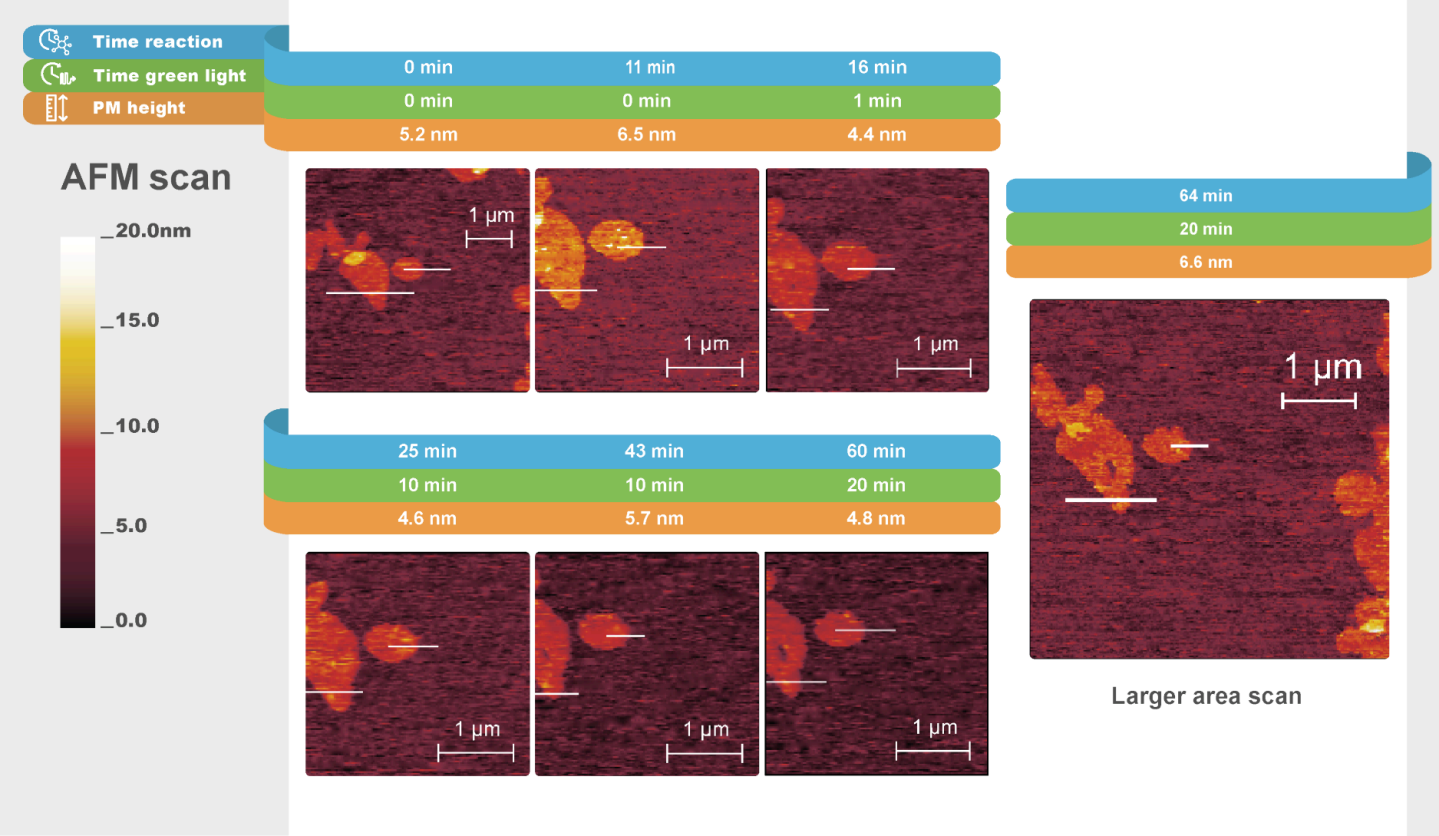
In situ AFM topography images (peakforce tapping mode in fluid) of hydrogel formation under the influence of PM (wild-type PM) and light at different times. The lines highlighted in white indicate the cross sections of PMs. The scan direction is from right to left, with a 0° scan angle. Cross section graphs are shown in Figure S7, Supporting Information.

Given the behavior of the WT and N-His_10_-tag PM in comparison to that of C-His_10_-tag PM in CLSM, the detection of fiber networks near the PM area during the liquid AFM scan was expected to be more challenging. The CLSM results indicate that gel formation rate for the C-His_10_-tag PM was at least 40 and 80 times faster than that for WT and N-His_10_-tag PMs, respectively (Figure 2). Indeed, the AFM scan of WT PMs (Figure 7) after the addition of reagents showed homogeneous PM patches before and after exposure to green light. Changes in relative height were observed over time. The most substantial change occurred when green light was applied to the sample after the addition of chemicals, resulting in a 2.1 nm decrease in patch height. However, over time, no changes in the shape or size of the PM patches were observed, and no new interfacial objects appeared (Figure S7, Supporting Information). The roughness of the AFM scan was approximately 0.5 nm, and the height difference ranged from 0.2 to 1.1 nm. The results strongly suggest that fiber growth at or near the WT-PM-aqueous interface did not take place upon exposure to light.

**Figure 8:**
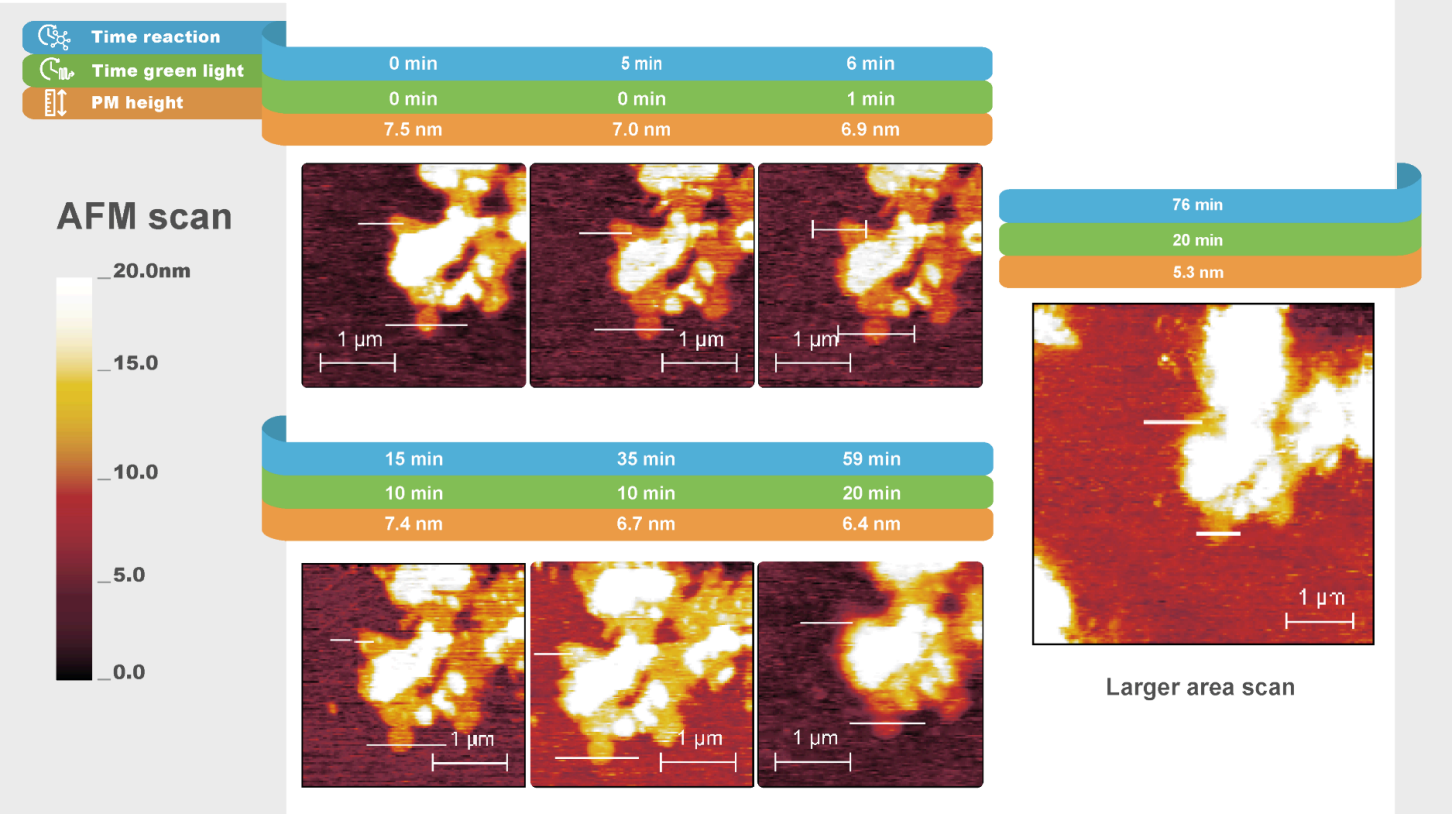
In situ AFM topography images (peakforce tapping mode in fluid) of hydrogel formation under the influence of PM (N-His_10_-tag PM) at different times. The lines highlighted in white indicate the cross sections of PMs. The scan direction is from right to left, with a 0° scan angle. Cross section graphs are shown in Figure S8, Supporting Information.

The AFM scan of N-His_10_-tag PMs (Figure 8) showed similar behavior to that of WT PMs. Following the addition of reagents, initially, homogeneous patches approximately 7.0 nm in height were observed before green light exposure. During all irradiation cycles, the relative height of the N-His_10_-tag PMs did not vary by more than 0.9 nm which we consider insignificant in respect of fiber growth. (Figure 8). Like with the WT PM, no changes to the shape and size of the PM patches were observed over time and no other interfacial objects appeared (Figure S8). Therefore, we concluded that fiber growth near the N-His_10_-tag PM aqueous interface did not occur upon exposure to light.

## Conclusions

This work demonstrates the possibility of using PM patches to spatiotemporally control the growth pattern of a self-assembled network of fibers. We expect that the formation of these structures can be used to detect the local formation of pH gradients, which in turn are controlled by the localization and orientation of PM patches and the presence of light. The results show that light-driven catalysts can be confined to produce patterned out-of-equilibrium gel materials. The experiments underscore the feasibility of employing an external stimulus, such as light, to direct self-assembly in synthetic systems through the spatial confinement of catalytic activity.

The results indicate that the presence and type of PMs significantly influence gelation rates. Depositing PMs on a negatively charged surface, CLSM showed faster in situ gel formation for C-His_10_-tag PMs, likely due to a favorably aligned pH gradient. AFM revealed that for C-His_10_-tag PMs, gels initially formed on PMs, and as protons spread, fibers grew around the PM area. The liquid AFM results were consistent with those obtained from CLSM, and both methodologies provided valuable insights into the effects of PM and light on our system. Specifically, CLSM effectively showed the sensitivity of our system to light, whereas liquid AFM illustrated the process of fiber formation in the vicinity of the PM area. The combination of CLSM and AFM introduced here provides a multifunctional toolbox for the optical imaging and characterization of spatiotemporal control of CRNs, which can be used to examine surface structures of fibers with high resolution. It may be used to identify and further characterize other CRNs and clarify the manner through which they dynamically assemble into functional domains. However, the identification of different fibers with higher resolution and scan rates remains a challenge. Moreover, for other CRNs, a more complex environment, such as apolar CRN products, can substantially increase the challenges of the experiment.

The strength of the formed hydrogel is a critical factor for its potential applications, particularly in biomaterials science. This study indicates that the components exhibit fast gelation under proton catalysis and stability of the hydrogel under physiological conditions. These properties make this platform highly promising for various biomedical applications, including tissue engineering and drug delivery. Furthermore, the ability to tune the hydrogel’s strength^33^ offers significant potential for biomaterials scientists exploring new materials for these fields. We anticipate that this study can underscore the potential for integrating synthetic self-assembly systems with living metabolic processes, opening avenues for innovative applications to enhance existing biological functions or develop entirely new ones.

Future studies should quantitatively measure the proton gradient based on the amount of light. This would enable the development of intelligent hydrogel networks at the nanoscale for application in sensing pH gradients.

## Supporting Information Available

Supplementary results of liquid AFM for the study of hydrogel formation (PDF)
