# Supplementary material for: Control of a Gel-Forming Chemical Reaction Network Using Light-Triggered Proton Pumps
Source: Langmuir. 2025 Mar 19;41(12):8071–80. doi: 10.1021/acs.langmuir.4c04581 (PMC11966745; doi:10.1021/acs.langmuir.4c04581)
Supplement: Supplementary file 1 — la4c04581_si_001.pdf [file la4c04581_si_001.pdf]

# Supplementary Information
# Control of a Gel-forming Chemical Reaction Network Using Light-triggered Proton Pumps

Jacqueline Figueiredo da Silva,[†,‡,§] Ardeshir Roshanasan,[‡,§] Marcel Bus,[‡]
Dimitrios Fotiadis,[¶] Armin W. Knoll,[†] Jan H. van Esch,[*,‡] and Heiko Wolf[*,†]

†*IBM Research Europe – Zurich, Säumerstrasse 4, 8803 Rüschlikon, Switzerland*

‡*Department of Chemical Engineering, Delft University of Technology, Van der Maasweg 9, 2629 HZ Delft, The Netherlands*

¶*Institute of Biochemistry and Molecular Medicine, University of Bern, 3012 Bern, Switzerland*

§*J.F.S and A.R. contributed equally to this work.*

E-mail: J.H.vanEsch@tudelft.nl; hwo@zurich.ibm.com

**Number of pages:** 8

**Number of figures:** 8

## Table of contents

Atomic Force Microscopy, S2

Liquid Atomic Force Microscopy, S3

## Atomic Force Microscopy

Figure S1 shows 20 $\times$ 20 $\mu m^2$ AFM scans at ambient conditions (Park Systems - NX20, contact mode tetrahedral cantilever) of different PM types. The blue circles indicate an example of single PM patches found under this technique. A single PM patch presented a height of 5 nm. A height higher than 10 nm indicates a PM agglomeration in the target area. The quantitative analysis of Figure S1 reveals that N-$His_{10}$-tag PMs presented the highest density of PM patches, covering 53.5 % of the area scanned. This is followed by wild-type PMs, with a coverage of 6.4 %, and then C-$His_{10}$-tag PMs, with a coverage of 4.5%.

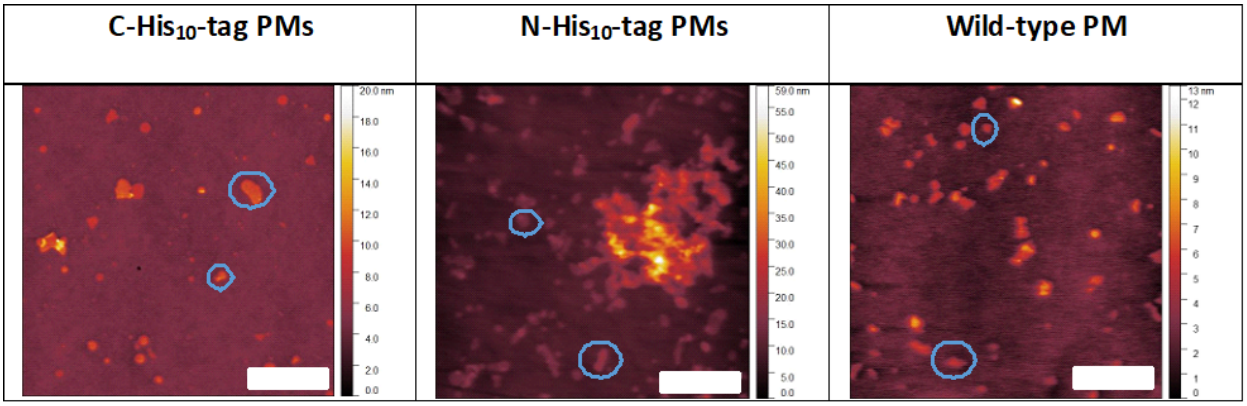


Figure S1: Topographical AFM images of deposited PMs (wild-type, C-$His_{10}$-tag, and N-$His_{10}$-tag PMs). Blue circles represent examples of single PM patches. Scale bar represents 5 µm.

## Liquid Atomic Force Microscopy

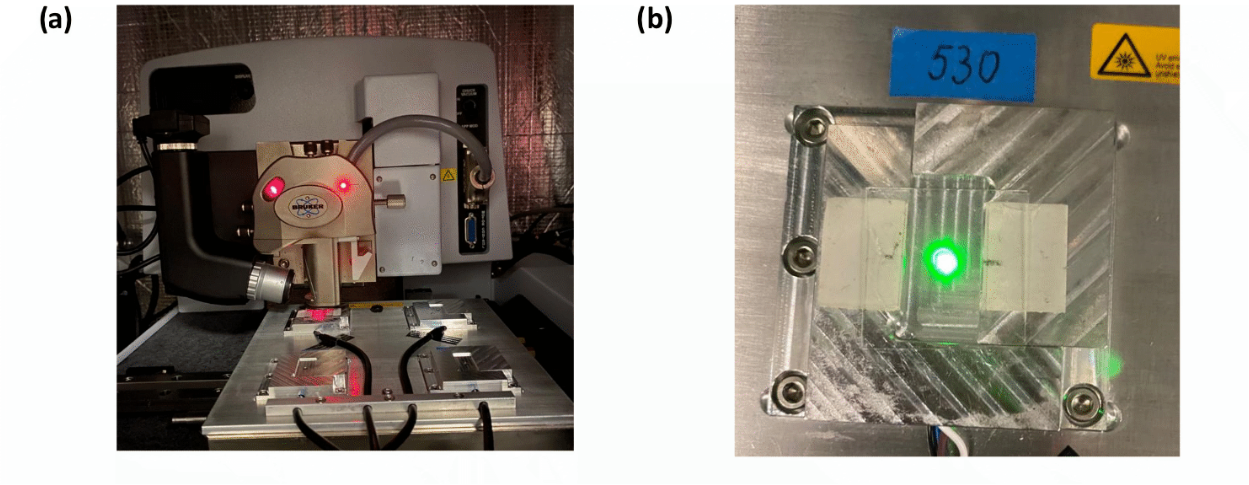


Figure S2: (a) Liquid AFM setup during a substrate scan. (b) 24 × 24 $mm^2$ glass sample exposed to green light by LED.

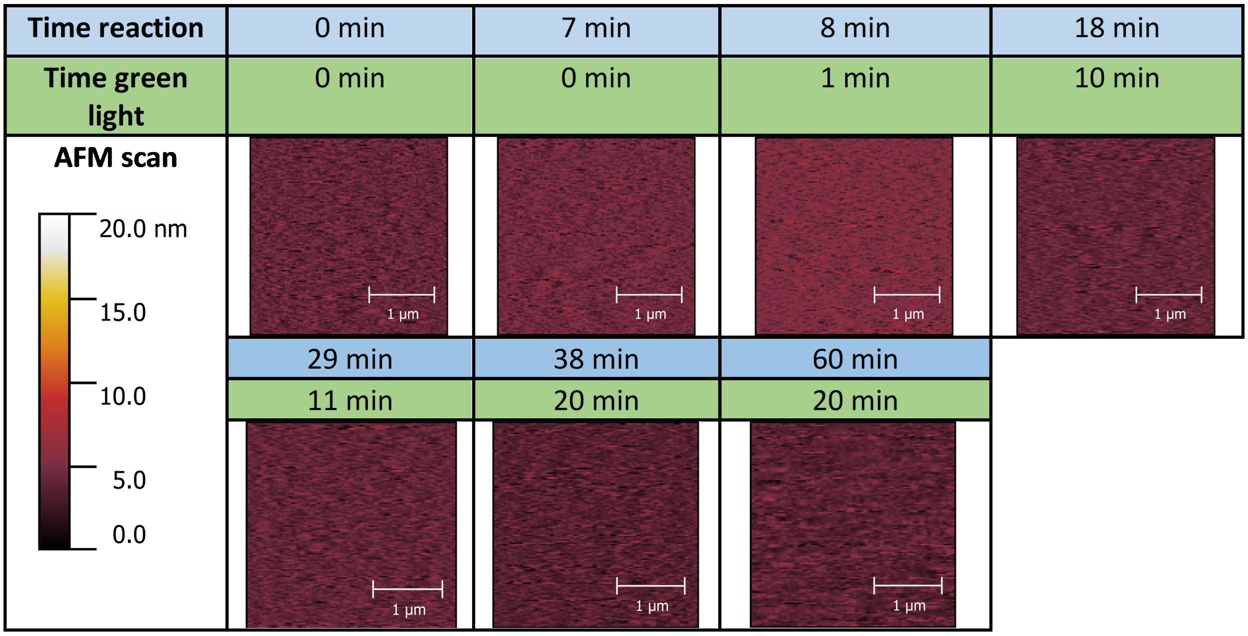


Figure S3: In-situ AFM topography images (Peakforce Tapping mode in fluid) of a $SiO_2$ substrate embedded with hydrogel reagents but without PM patches, under exposure to green light at various time intervals. The scan direction is from right to left, with a 0° scan angle.

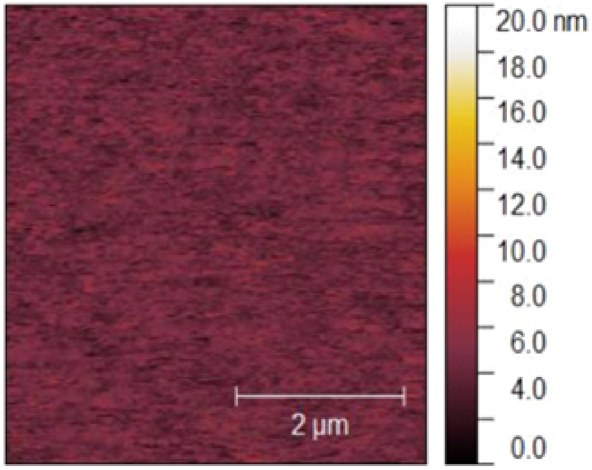


Figure S4: In-situ AFM topography images (Peakforce Tapping mode in fluid) of a $SiO_2$ substrate embedded with hydrogel reagents, but devoid of PM patches, exposed to green light at the conclusion of the experiment. The scan was conducted over a 5 $\times$ 5 μ$m^2$ area, with the scanning direction oriented from right to left and at a 0° scan angle.

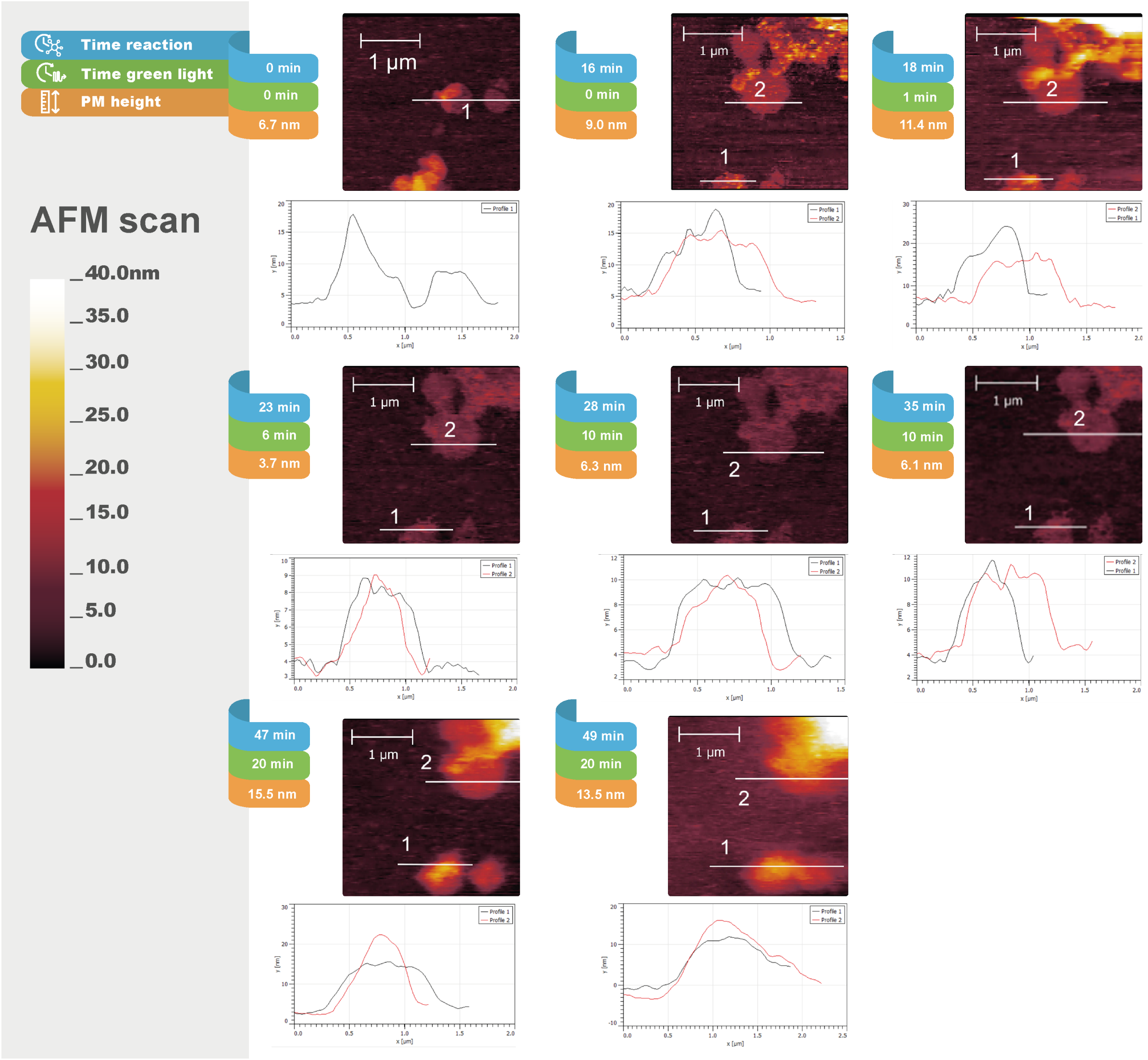


Figure S5: Height cross sections of hydrogel formation under the influence of PMs (C-$His_{10}$-tag PMs) and light at different time points, presented in Figure 4 (main text).

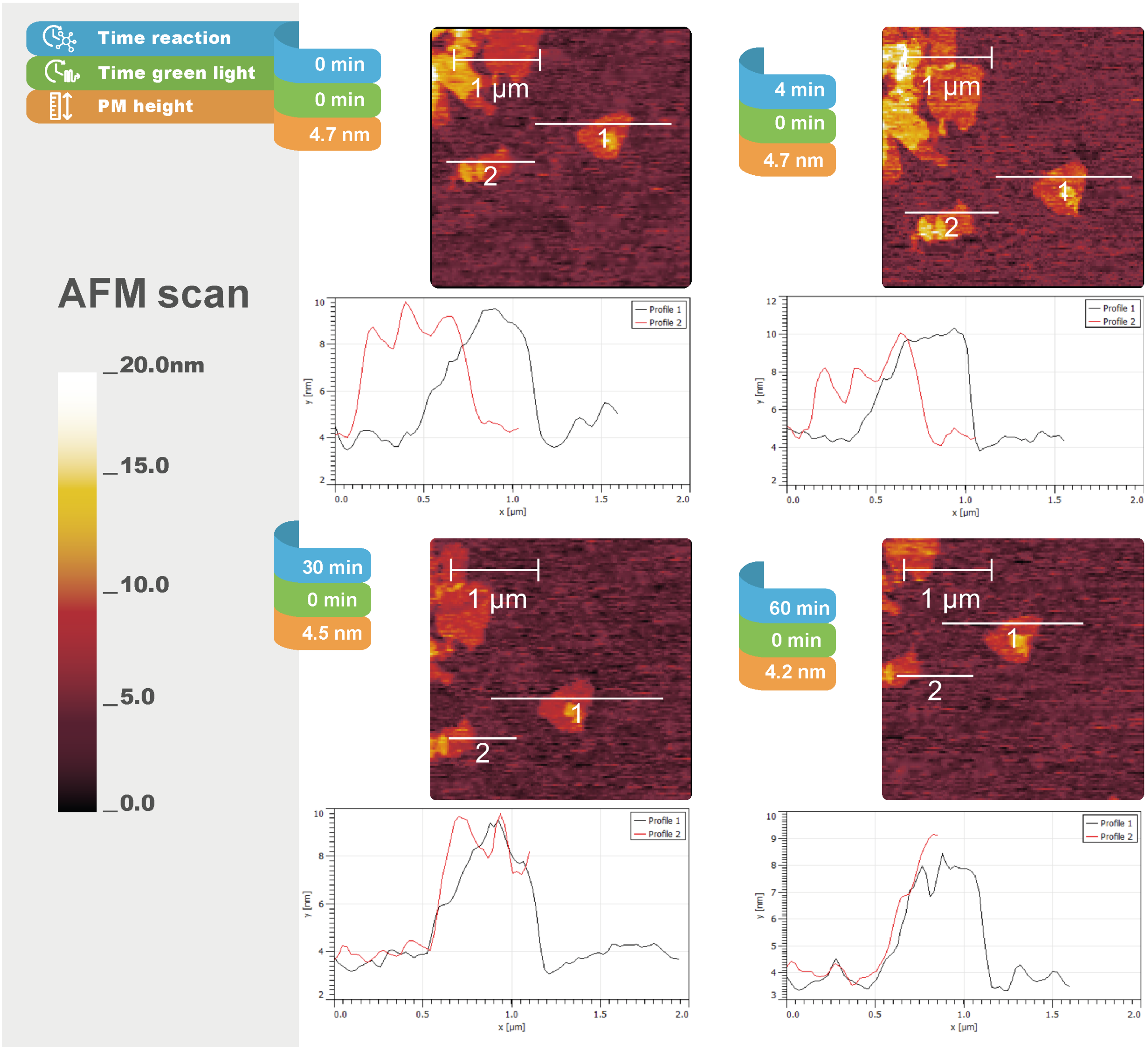


Figure S6: Height cross sections of hydrogel formation under the influence of PMs (C-His$_{10}$-tag PMs) in the dark at different time points, presented in Figure 5 (main text).

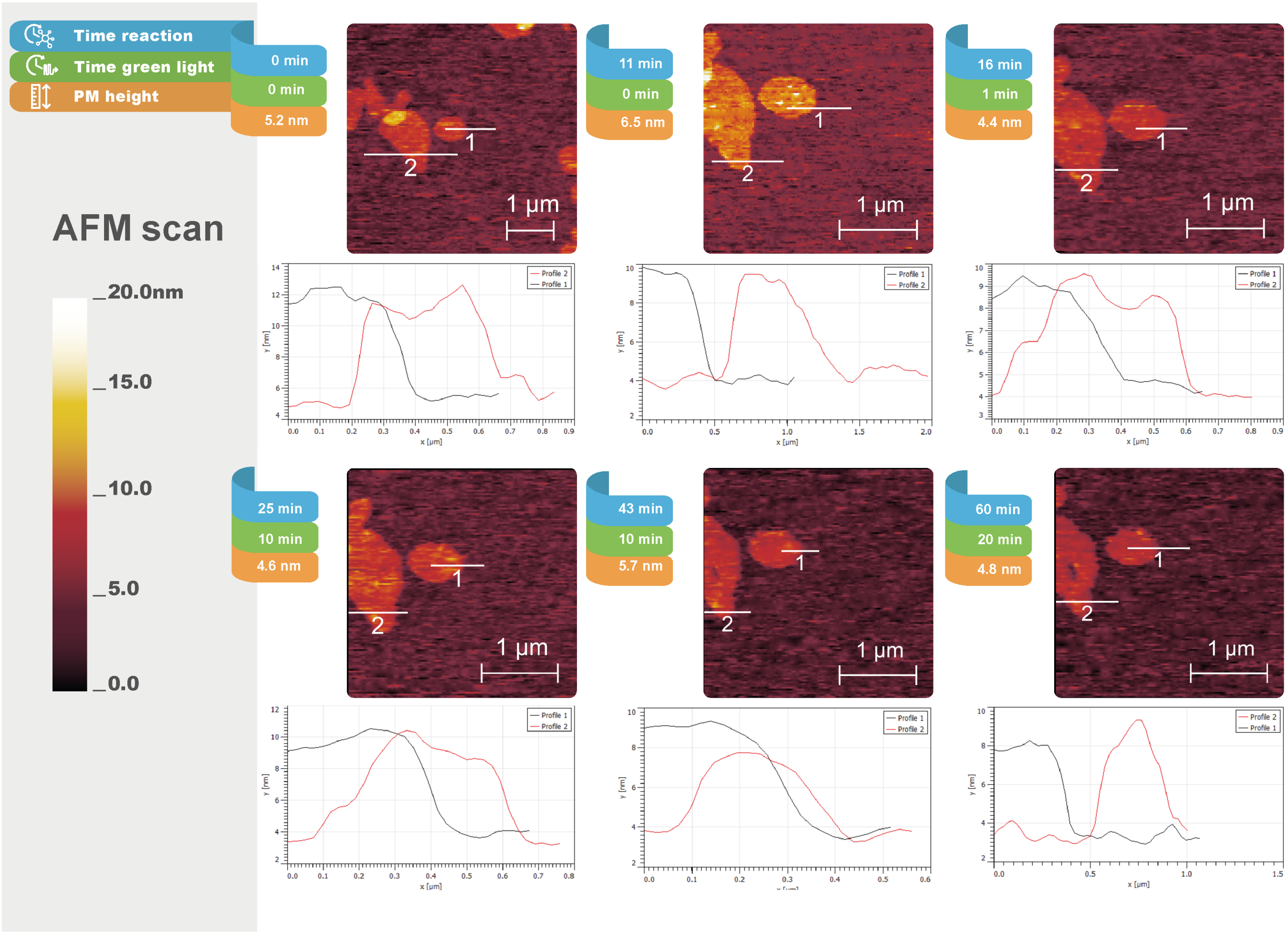


Figure S7: Height cross sections of hydrogel formation under the influence of PMs (wild type PMs) and light at different time points, presented in Figure 7 (main text).

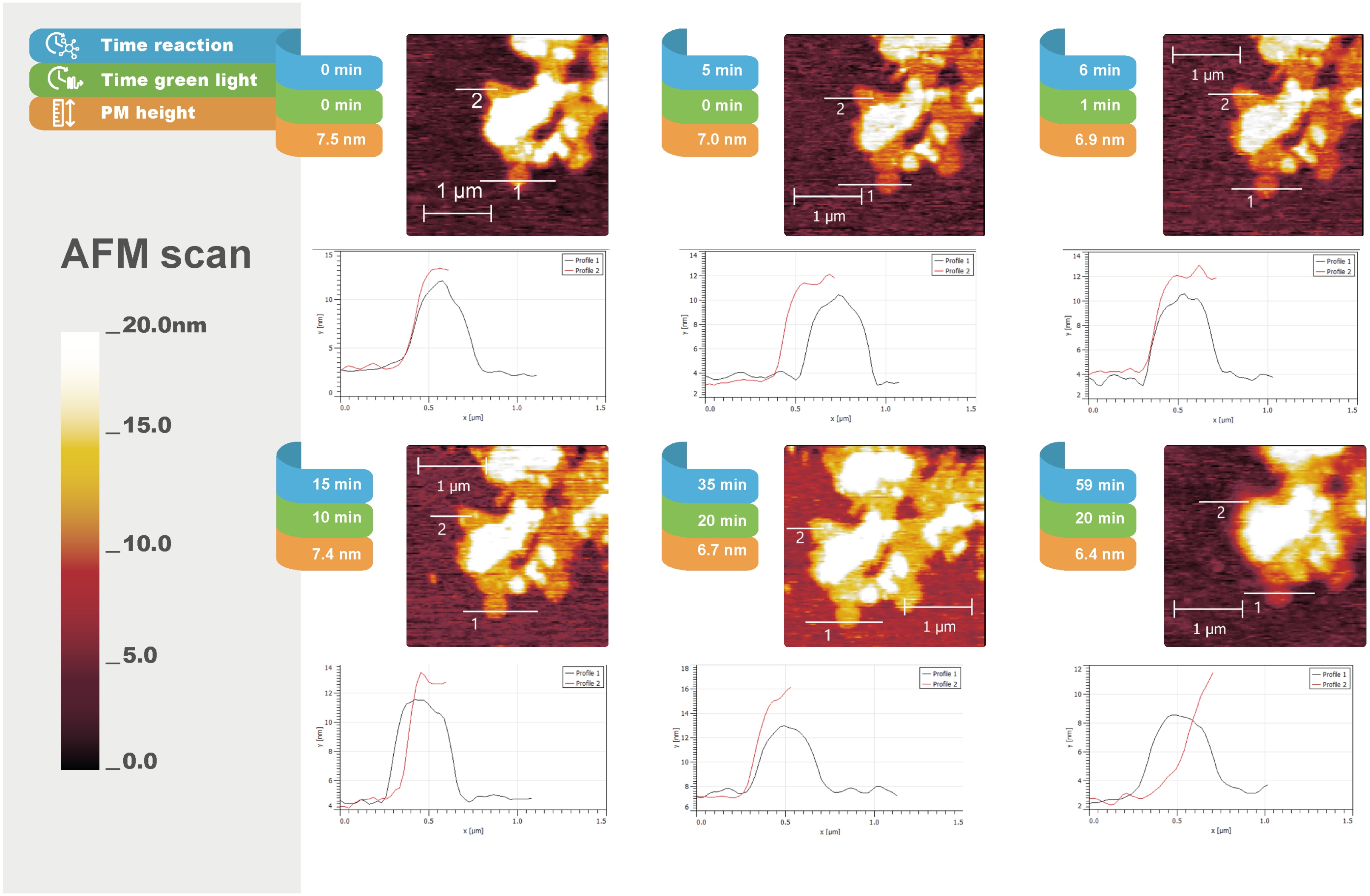


Figure S8: Height cross sections of hydrogel formation under the influence of PMs (N-His$_{10}$-tag PMs) and light at different time points, presented in Figure 8 (main text).
